# Phenolic Acids and Prevention of Cognitive Decline: Polyphenols with a Neuroprotective Role in Cognitive Disorders and Alzheimer’s Disease

**DOI:** 10.3390/nu14040819

**Published:** 2022-02-15

**Authors:** Giuseppe Caruso, Justyna Godos, Anna Privitera, Giuseppe Lanza, Sabrina Castellano, Alessio Chillemi, Oliviero Bruni, Raffaele Ferri, Filippo Caraci, Giuseppe Grosso

**Affiliations:** 1 Department of Drug and Health Sciences, University of Catania, 95125 Catania, Italy; forgiuseppecaruso@gmail.com (G.C.); annaprivitera01@gmail.com (A.P.); 2 Research Operative Unit of Neuropharmacology and Translational Neurosciences, Oasi Research Institute—IRCCS, 94018 Troina, Italy; 3 Department of Biomedical and Biotechnological Sciences, University of Catania, 95123 Catania, Italy; justyna.godos@gmail.com (J.G.); alessio.chillemi90@gmail.com (A.C.); giuseppe.grosso@unict.it (G.G.); 4 Clinical Neurophysiology Research Unit, Oasi Research Institute—IRCCS, 94018 Troina, Italy; glanza@oasi.en.it; 5 Department of Surgery and Medical-Surgical Specialties, University of Catania, 95123 Catania, Italy; 6 Department of Educational Sciences, University of Catania, 95124 Catania, Italy; sabrina.castellano@unict.it; 7 Department of Developmental and Social Psychology, Sapienza University, 00185 Rome, Italy; oliviero.bruni@uniroma1.it; 8 Sleep Research Centre, Department of Neurology IC, Oasi Research Institute—IRCCS, 94018 Troina, Italy; rferri@oasi.en.it

**Keywords:** polyphenols, cognitive status, Alzheimer disease, neurodegeneration, neuroprotection, secondary prevention

## Abstract

Cognitive impairment, also known as cognitive decline, can occur gradually or suddenly and can be temporary or more permanent. It represents an increasingly important public health problem and can depend on normal aging or be linked to different neurodegenerative disorders, including Alzheimer’s disease (AD). It is now well-established that lifestyle factors including dietary patterns play an important role in healthy aging as well as in the prevention of cognitive decline in later life. Among the natural compounds, dietary polyphenols including phenolic acids have been recently the focus of major attention, with their supplementation being associated with better cognitive status and prevention of cognitive decline. Despite their therapeutic potential, human studies investigating the relation between phenolic acids intake and cognitive outcomes are rather scarce. In this review, we provide preclinical evidence that different dietary polyphenols such as rosmarinic acid, ellagic acid, and cinnamic aldehyde can exert neuroprotective and pro-cognitive activities through different molecular mechanisms including the modulation of pro-oxidant and antioxidant machinery as well as inflammatory status. Future and more numerous in vivo studies are needed to strengthen the promising results obtained at the preclinical level. Despite the excellent pharmacokinetic properties of phenolic acids, which are able to be accumulated in the brain at pharmacologically relevant levels, future studies should also identify which among the different metabolites produced as a consequence of phenolic acids’ consumption may be responsible for the potential neuroprotective effects of this subgroup of polyphenols.

## 1. Introduction

Growing evidence supports the hypothesis that dietary factors may play a role in healthy aging, including a protective effect against age-related cognitive decline [1]. A recent summary of the literature showed that dietary polyphenols have been associated with better cognitive status and their supplementation may prevent cognitive decline [2].

The most common group of polyphenolic compounds is represented by flavonoids and the main subclasses flavonols, flavones, flavanones, flavan-3-ols, anthocyanidins, and isoflavones, largely contained in fruits and vegetables [3]. The potential of these molecules in preventing and/or counteracting neurodegenerative phenomena has been recently considered [4,5,6]. Other classes of polyphenols largely investigated for their potential role in neurodegenerative diseases include stilbenes (such as resveratrol, contained in red wine), tyrosol (such as oleuropein, contained in olive oil), and curcuminoid (such as curcumin, contained in turmeric) [2]. Finally, another non-flavonoid group of polyphenols includes phenolic acids, a heterogeneous family of polyphenols commonly consumed, largely underrated, and far less investigated concerning their potential effects against neurodegenerative diseases [2]. From a general point of view, phenolic acids may exert a number of neuroprotective and cognition-enhancing effects related to the anti-amyloidogenic and anti-aggregant activity of these natural compounds [2]. Moreover, phenolic acids have been demonstrated to inhibit the aggregation of proteins involved in the pathogenesis of various neurodegenerative pathologies characterized by cognitive deterioration, including Alzheimer’s disease (AD), Parkinson’s disease (PD), dementia with Lewy bodies, and multiple system atrophy [7,8,9]. Thus, there is a growing body of literature on preclinical studies and experimental models showing a mechanistic rationale for the application of polyphenols against cognitive impairment, with possible use in the future toward more advanced and disabling dementias, including AD.

Recently, neuroprotective [10] and cognition-improving [11,12] effects of this class of natural compounds have been reviewed. However, there is currently no updated summary providing an overview of the studies focused on cognitive outcomes. The aim of this study is to review current evidence from human studies on phenolic acid intake and cognitive status, including cognitive impairment and dementias, as well as provide an overview of preclinical mechanistic studies on these compounds on neurodegeneration and other mechanisms leading to cognitive disorders.

## 2. Chemical Composition, Major Groups, and Dietary Sources of Phenolic Acids

Phenolic acids (or phenolcarboxylic acids) are aromatic acid compounds containing a phenolic ring and an organic carboxylic acid function [3]. The main groups composing this class of polyphenols are hydroxycinnamic acids and hydroxybenzoic acids. Hydroxycinnamic acids are chemically characterized by a structure constituted of a nine carbon (C6-C3) skeleton and a side chain double bond characterized by a *cis* or *trans* configuration. Among the most studied molecules belonging to this group, caffeic, chlorogenic, o-coumaric, p-coumaric, m-coumaric, ferulic, and cinnamic acids are the most commonly consumed in the human diet being contained in coffee [13]. Hydroxybenzoic acids include in their structure a C6-C1 carbon backbone with methoxylations and hydroxylations at the aromatic ring: the main molecules of interest include gallic, p-hydroxybenzoic, vanillic, syringic, and protocatechuic acids; the main dietary sources of these compounds are certain cereals (i.e., bran, grain brown rice, and derivatives, such as beer), olive oil, tea (despite some differences between types and fermentation), some fruits (i.e., cherries, plums, and gooseberries among others), and red wine [13].

## 3. Studies Conducted on Humans on Phenolic Acids and Cognitive Status

Studies investigating the association between phenolic acid intake and cognitive outcomes are rather limited. The first published study was conducted on the SU.VI.MAX (“Supplémentation en Vitamines et Minéraux Antioxydants”) study including 2574 middle-aged adults (mean age 66 years old) participating in the cohort assessed in 1994–1996 for cognitive function with four neuropsychological tests (phonemic and semantic fluency, the RI-48 Cued Recall test, the Trail Making test, and Forward and Backward Digit Span) and followed up to 2007–2009; the authors reported that intake of hydroxybenzoic acids was positively associated with language and verbal memory (especially with episodic memory; P = 0.0004) [14]. After being unexplored for several years, a recent study conducted on the SUN (“Seguimiento Universidad de Navarra”) Project, a Spanish prospective cohort study from which a subsample of 806 older participants (mean age 66 years old) was investigated for cognitive function through the validated Spanish Telephone Interview for Cognitive Status-modified score, showed no association between phenolic acid intake and cognitive status [15]. Finally, a recent study conducted by our group supports a protective effect of phenolic acid intake against cognitive decline. The study involved 883 older individuals (mean age 64.9 years old, 56.7% female) from the MEAL (Mediterranean healthy Eating, Aging, and Lifestyle) study recruited from the general population of Catania, South Italy, showed an inverse association between the highest quartile of total phenolic acid intake and impaired cognitive status [odds ratio (OR) = 0.36, 95% confidence interval (CI): 0.14 to 0.92] [16]. Among specific groups and individual compounds, significant associations were found for greater intake of hydroxycinnamic acids (OR = 0.35, 95% CI: 0.13 to 0.91) and caffeic acid (OR = 0.32, 95% CI: 0.11 to 0.93) [16].

As previously mentioned, the most common dietary sources of phenolic acids, on which some studies have been conducted concerning cognitive outcomes, include coffee, tea, red wine, and olive oil. A recent meta-analysis showed that moderate alcohol (<11 g/day) and coffee (<2.8 cups/day) intake was associated with a lower risk of cognitive deficits or only dementia [17]. Moreover, the same study reported an inverse linear association between green tea consumption and cognitive impairment (1 cup/day, relative risk = 0.94; 95% CI: 0.92 to 0.97) [17].

## 4. Preclinical Studies on Phenolic Acids and Cognitive Disorders: Molecular Mechanisms and Neuroprotective Activity

**Figure 1:**
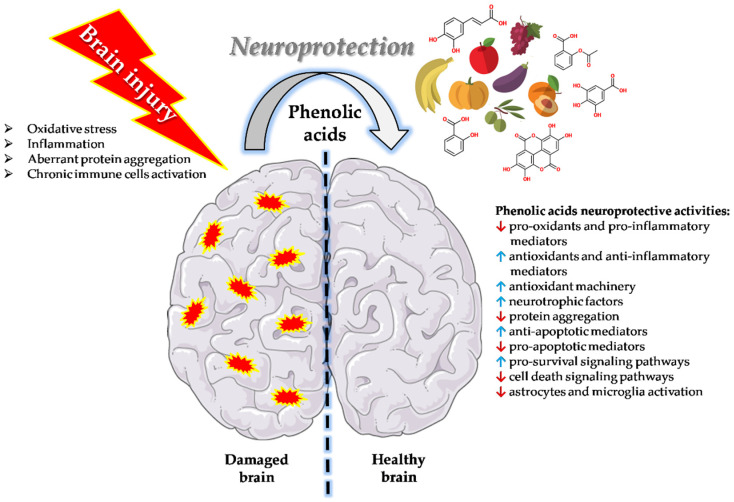
Phenolic acids neuroprotective activities. Phenolic acids can exert neuroprotection through numerous mechanisms including the ability to decrease the levels of pro-oxidants and pro-inflammatory mediators, increase the production and/or the activity of antioxidants and anti-inflammatory mediators, enhance the antioxidant machinery, rescue and/or increase the release of neurotrophic factors, prevent and/or counteract protein aggregation, increase the expression of anti-apoptotic mediators and down-regulate that of pro-apoptotic mediators, activate pro-survival signaling pathways also blocking cell death signaling pathways, and decrease the activity of reactive astrocytes and microglia.

Phenolic acids can exert neuroprotective and pro-cognitive activities through numerous mechanisms that may differ across the various natural compounds part of this heterogeneous group (Figure 1).

### 4.1. Caffeic Acid and Caffeic Acid Phenethyl Ester

Recent evidence suggests that caffeic acid exerts neuroprotective effects through modulating neuroinflammation and oxidative stress. In particular, caffeic acid has been shown to attenuate lipopolysaccharide (LPS)-induced sickness behaviour and neuroinflammation in mice [18]. This phenolic acid, administered orally (30 mg/kg) one hour prior to LPS (1.5 mg/kg), reduced in a dose-dependent manner the levels of the inflammation marker tumor necrosis factor-alpha (TNF-α) in the serum. Additionally, caffeic acid administration rescued the antioxidant defence system via a significant decrease of malondialdehyde (MDA; one of the final products of polyunsaturated fatty acids peroxidation in the cells [19]) and rescue of glutathione (GSH; a major tissue antioxidant [20]) levels in the brain. Although the pathophysiology of PD is not yet clearly understood, the evidence suggests its multifactorial nature. An aberrant aggregation of a neuronal protein, namely α-synuclein (α-syn), has been recognized among key contributors to the process of neurodegeneration and PD pathophysiology. Interestingly, an inhibitory effect of caffeic acid against an antidepressant-induced fibrillogenesis of human α-syn in a concentration-dependent manner was proved [21], suggesting the design of new therapeutic drugs for PD based on caffeic acid structure. In an in vivo study employing an epilepsy mouse model, caffeic acid showed neuroprotective action against oxidative and DNA damage [22]. In a different in vivo study, caffeic acid at two different doses (4 or 8 mg/kg) was able to reduce the latency to sleep in the diazepam-induced sleeping time test as well the genotoxic damage induced by aspilocarpine in an acute seizure model of mouse [23]. Chang et al. demonstrated the protective effect of caffeic acid against AD through the modulation of cerebral insulin signaling, amyloid-β (Aβ) accumulation, and synaptic plasticity in a hyperinsulinemic rat model [24]; in particular, the oral administration of caffeic acid (30 mg/kg) significantly ameliorated memory and learning impairments, enhanced superoxide dismutase (SOD) and glutathione free radical scavenger activity, increased the levels of p-glycogen synthase kinase-3 beta (GSK-3β)(Ser9) and decreased the expression of p-tau protein in the hippocampus, attenuated the expression of amyloid precursor protein (APP) and β-site APP cleaving enzyme, and increased the expression of synaptic proteins in this rat model.

Caffeic acid phenethyl ester has shown to be neuroprotective in rats exposed to ionizing radiation by decreasing radiation-induced oxidative damage through the amelioration of SOD activity and the decrease in MDA levels in the brain [25]. This ester of caffeic acid has also been shown to exert neuroprotection through the modulation of different pathways such as nuclear factor erythroid 2–related factor 2 (Nrf2), nuclear factor kappa-light-chain-enhancer of activated B cells (NF-κB), and signal transducer and activator of transcription 3 (STAT3) [26] along with 3-kinase/protein kinase B, Bax and BAD, Bcl-2 and Bcl-XL, and mitogen-activated protein kinase (MAPK) [27]. The protective effects of caffeic acid phenethyl ester, injected intraperitoneally (i.p.), against ifosfamide (IFOS)-induced central neurotoxicity in rats was mediated by its ability to decrease MDA and protein carbonyl levels at brain level [28,29]. In vitro, caffeic acid phenethyl ester protected PC12 cells from 1-methyl-4-phenylpyridinium (MPP+)-induced cell death by increasing the network of neurites as well as the expression of proteins responsible for axonal growth (GAP-43) and synaptogenesis (synaptophysin and synapsin I) [30]. Caffeic acid phenethyl ester also possesses antioxidant activity, as demonstrated in a work carried out by Tsai et al. in which it significantly inhibited the expressions of inducible nitric oxide synthase (iNOS), cyclooxygenase (COX)-2, and the production of nitric oxide (NO) in LPS-activated microglial cells [27]; these effects were paralleled by the attenuation of LPS-dependent MAPK and Akt signaling pathways and the induction of heme oxygenase-1 (HO-1) and erythropoietin (EPO). Lastly, caffeic acid phenethyl (30 mg/kg) has shown to be neuroprotective by counteracting 3-nitropropionic acid-induced striatal neurotoxicity, representing a model of Huntington’s disease (HD) [31]; it reduced striatal damage and the behavioral deficits also reducing the activation of astrocyte (glial fibrillary acidic protein; GFAP) and microglia (CD45).

### 4.2. Chlorogenic Acid

Chlorogenic acid has been demonstrated to protect primary neurons against glutamate neurotoxicity by regulating the intracellular concentrations of Ca(2+) [32]. This polyphenol has also shown its neuroprotective and anti-inflammatory potential in microglial cells infected with herpes simplex virus [33]; in these infected cells, chlorogenic acid increased the survival rate, prevented the increase in toll-like receptor 2 (TLR2), TLR9, and Myd88, attenuated TNF-α and interleukin (IL)-6 release, and reduced the expression of NF-κB p65. Chlorogenic acid-enriched extract from Eucommia ulmoides showed an antidepressant potential in vivo paralleled by neuroprotection and promotion of serotonin release through the enhancement of synapsin I expression in vitro [34]. Chlorogenic acid also possesses antioxidant activity; in fact, it was able to decrease MDA and reactive oxygen species (ROS) levels in hydrogen peroxide (H_2_O_2_)-induced alterations in rat brain slices [35]. In a different study, the expression pattern of N-methyl-D-aspartate (NMDA) receptors revealed an antiepileptic potential of chlorogenic acid in a pilocarpine-induced epileptic mouse model [36]. The administration of chlorogenic acid (5 mg/kg) reduced seizures through the reduction of lipid peroxidation and nitrite content, as well as the mRNA expressions of NMDA receptors, metabotropic glutamate receptor 1 (mGluR1) and mGluR5 in the hippocampus. The beneficial effects of chlorogenic acid were also observed in the case of PC12 cells challenged with ethanol [37]. In this experimental model, the polyphenol increased the cell viability, promoted the proliferation of damaged cells, increased the distribution ratio of the cells at the G2/M and S phases, enhanced mitochondrial transmembrane potential, up-regulated the expression of Bcl-2 and GAP-43, and down-regulated the expression of caspase-3. A study carried out by Taram et al. investigated the neuroprotective effects of chlorogenic acid and its major metabolites in primary cultures of rat cerebellar granule neurons [38]. Chlorogenic and caffeic acids displayed a relevant neuroprotective effect against the NO donor, sodium nitroprusside, while caffeic acid and ferulic acid significantly protected neurons against glutamate-induced cell death. It is worth highlighting that caffeic acid was the only compound to display significant protection against H_2_O_2_ stimulation, inhibition of proteasome, caspase-dependent intrinsic apoptosis, and endoplasmic reticulum stress.

### 4.3. Ferulic Acid

The neuroprotective activity of ferulic acid often depends on the combination of its antioxidant and anti-inflammatory properties. Ferulic acid was able to increase serotonin and norepinephrine levels, but not those of dopamine, in the mouse hippocampus and frontal cortex, brain regions that play a key role in the pathophysiology of mood disorders [39]. It also reduced depressive-like behavior measured as tail suspension and forced swim tests (TST and FST); the inhibition of monoamine oxidase A (MAO-A) activity coming from ferulic acid treatment was also demonstrated in this study. The antioxidant defense system plays a pivotal role in the antidepressant-like effect exerted by ferulic acid. In fact, improvement of TST and FST scores has been paralleled by the increased expression of SOD, catalase (CAT), and glutathione peroxidase (GPx) in the cerebral cortex of mice [40]. SOD and CAT were also positively modulated in a rotenone (ROT)-induced rat model of PD [41], in which ferulic acid was also able to rescue dopaminergic neurons in substantia nigra pars compacta area and nerve terminals in the striatum, restore glutamate levels, prevent lipid oxidation, reduce the levels of ionized calcium-binding adapter molecule (Iba-1), GFAP, pro-inflammatory cytokines, COX-2, and iNOS. A combination of antioxidant and anti-inflammatory activities of ferulic acid was also observed in microglial cells stimulated with LPS (inhibition of TNF-α, IL-6, IL-1, and NO; reduction of COX-2 and iNOS) [42] and Neuro-2a cells challenged with H_2_O_2_ (up-regulation of brain-derived neurotrophic factor (BDNF) gene and down-regulation of nNOS, eNOS, COX-2, IL-1β, caspase-9, and BCL-2 genes) [43]. A significant up-regulation of the BDNF, postsynaptic density protein (PSD95), and synapsin I levels was also observed in the prefrontal cortex and hippocampus of a chronic model of depression (i.e., chronic unpredictable mild stress) [44]. PC12 cells are often used as a cell model to study the neuroprotective features of candidate molecules. In this cell line, ferulic acid dose-dependently inhibited the LPS-induced production of TNF-α and IL-1β and attenuated the LPS-induced up-regulation of phosphodiesterase 4 (PDE4) activity; furthermore, ferulic acid decreased the up-regulation of the PDE4B mRNA and reversed the down-regulation of cAMP-response element binding protein (CREB) and pCREB induced by LPS [45]. The modulation of the MAPK kinase (MEK)/ extracellular signal-regulated kinases (ERK)/p90 ribosomal S6 kinases (p90RSK) signaling pathway, and the related neuroprotective activity, was observed instead in an in vivo model of focal cerebral ischemic injury [46]. With regard to its anti-apoptotic activity, increased cell viability, prevented membrane damage, scavenged free radicals, increased the activity of SOD, decreased intracellular free Ca(2+) levels, lipid peroxidation, and prostaglandin E2 (PGE2) production in hypoxia-stressed PC12 cells. Ferulic acid also reduced p-p38 MAPK, caspase-3, and COX-2 activation [47].

### 4.4. Gallic Acid

Gallic acid is a phenolic acid that may be effective against age-related and disease-related cognitive decline, because of its well-documented antioxidant, anti-inflammatory, and neuroprotective activities. From a mechanistic perspective, gallic acid has been reported to decrease the level of cytokines in microglia cells and protect neurons from Aβ-induced neurotoxicity by inhibiting NF-κB acetyltransferase [48]. Interestingly, in the same study, it was also observed that gallic acid counteracted Aβ-induced cognitive dysfunction in mice [48]. In line with these findings, another preclinical study reports that administration of gallic acid prevented memory deficits and synaptic impairment by suppressing the release of the pro-inflammatory cytokines IL-1β, IL-6, and TNF-α [49]. Moreover, it was found that chronic administration of gallic acid rescued learning and memory deficits of Aβ-protein precursor/presenilin 1 (APP/PS1) transgenic mice, an animal model of AD [50]. In particular, this beneficial effect was linked to reduced neuroinflammation in terms of reduced Aβ plaque-associated microgliosis and astrocytosis [50]. In this study, the authors further show that gallic acid is able to behave as a dual α/β-secretase modulator, consequently affecting APP processing, and that can promote APP cleavage by α-secretase through increasing the activity of the disintegrin and metalloproteinase domain-containing protein 10 (ADAM10). This leads to an accumulation of soluble APP-α protein, which has been found to have neurotrophic and neuroprotective activities [51] and to enhance synaptic plasticity [52]. Similarly, the pro-cognitive effect of gallic acid as well as its beneficial effect on synaptic impairment were further observed and associated with a disrupting action of this small molecule on Aβ1-42 aggregation [53]. Furthermore, it has been reported that tannic acid, a hydrolysable glycosidic polyphenol polymer of gallic acid, has neuroprotective, anti-inflammatory, and pro-cognitive properties. It indeed prevented streptozotocin (STZ)-induced memory impairment, and restored the increased level of the pro-inflammatory cytokines IL-6 and TNF-α as well as the decreased level of Akt and pAkt [54]. Gallic acid also showed neuroprotective effects in a rat model of traumatic brain injury (TBI) ameliorating memory and long-term potentiation (LTP) impairment through the decrease in brain lipid peroxidation (MDA) and pro-inflammatory cytokines (IL-1β, IL-6, and TNF-α) in the brain.

The neuroprotective effects of gallic acid against STZ-induced oxidative damage in rat striatum have been demonstrated [55]. In this animal model, the oxidative damage was counteracted by the chronic administration of gallic acid (30 mg/kg for 26 days) that normalized thiobarbituric acid reactive substances (TBARS) and total thiol contents, as well as the activity of the antioxidant enzymes GPx, CAT, and SOD activities in the rat striatum. Still in the context of the antioxidant activity, gallic acid, orally administered at the dose of 60 and 120 mg/kg, was able to ameliorate cyclophosphamide-induced neurotoxicity in Wistar rats through free radical scavenging activity and the rescue of normal levels of cerebellar and cerebral CAT, SOD, MDA, glutathione S-transferase (GST), GPx, and nitrite [56]. A very recent work investigated the effects of gallic acid against sodium arsenite-induced neurotoxicity in rats [57]. In this rat model, gallic acid significantly reversed sodium arsenite-induced reduction of step-through latency, latency to fall, and crossing, rearing, and grooming activity, being also able to reduce MDA and increase GSH levels and GPx activity in different regions of the brain. Lastly, nutritional supplementation of gallic acid (100 mg/kg) ameliorates AD-type hippocampal neurodegeneration and cognitive impairment (spatial memory and learning) induced by aluminum chloride (AlCl_3_) exposure in adult Wistar rats [58]. In more detail, following AlCl_3_ exposure there was a significant decrease in CAT, GSH, and SOD, as well as serum electrolyte and neurotransmitter levels with a corresponding increase in MDA, hydrogen peroxide (H_2_O_2_), and NO that was restored nearly to normal after gallic acid administration.

### 4.5. Rosmarinic Acid

The ability of rosmarinic acid (4 mg/kg) to reduce the levels of free radicals and DNA damage in the kindling CF-1 mouse model of epilepsy pentylenetetrazole (PTZ)-induced has been demonstrated [22]. This molecule was also shown to increase the latency and decrease the percentage of seizure incidents in the same mouse model. In a different study employing the same animal model, the same dosage of rosmarinic acid coupled to diazepam improved the latency to first seizures, also reducing the latency to sleep in the diazepam-induced sleeping time test. The same study also reported the ability of rosmarinic acid (2 or 4 mg/kg) to decrease pilocarpine-induced genotoxic damage in a mice acute seizure model [23]. The neuroprotective activity exerted by rosmarinic acid could also depend on its modulatory activity on the Nrf2 pathway. In fact, as shown by Fetoni et al., the i.p. administration of rosmarinic acid (10 mg/kg) in male adult Wistar rats attenuated noise-induced hearing loss and hair cell damage, reduced the ROS imbalance induced by noise as well as superoxide generation and lipid peroxidation, and induced HO-1 up-regulation through the activation of Nrf2-antioxidant-responsive element (ARE) [59]. In a study employing C6 glial cells, rosmarinic acid was able to increase cell viability by decreasing both oxidative stress and lipid peroxidation H_2_O_2_-induced [60]. In the same H_2_O_2_-stimulated cells, rosmarinic acid was able to reduce the gene and protein expression levels of iNOS and COX-2. The antioxidant power of rosmarinic acid, characterized by the reduction in the levels of ROS and reactive nitrogen species (RNS), the inhibition of lipid peroxidation, along with the inhibition of monoamine oxidases (MAO-A and MAO-B) and catechol-O-methyltransferase (COMT) enzymes, has also been demonstrated [61]. Of note, no cytotoxicity was observed on polymorphonuclear rat cells, even if used at a concentration higher than those displaying the antioxidant profile and enzyme inhibition effects. Spinal cord injury (SCI) has been linked to loss of neuronal function. In rats with SCI, the i.p. administration of rosmarinic acid (10 mg/kg) significantly enhanced the antioxidant status, decreased oxidative stress, and down-regulated NF-κB and pro-inflammatory cytokines [62]. Stress represents a risk factor of AD, and it has been shown that stress could induce tau phosphorylation and increase tau insolubility in the brain. In a chronic restraint stress mouse model, rosmarinic acid was able to counteract the stress-induced tauopathy by decreasing p-tau and insoluble p-tau formation and reverting the abnormal changes of chaperones and peptidyl-prolyl *cis*/*trans* isomerase (Pin1) [63]. As shown by Hwand et al., the acute rosmarinic acid treatment enhances LTP, BDNF, and AMPA glutamate receptor 2 (GluR-2) protein expression, and cell survival rate against scopolamine challenge in rat organotypic hippocampal slice cultures [64]. Lastly, rosmarinic acid exerted neuroprotection in a neuropathic pain animal model by decreasing the levels of different spinal pro-inflammatory markers such as COX2, PGE2, IL-1β, matrix metallopeptidase 2 (MMP2), and NO [65].

### 4.6. Acetylsalicylic Acid

Acetylsalicylic acid is a frequently used drug for the treatment of inflammatory pain and fever. The neuroprotective activity of acetylsalicylic acid (50 mg/kg) has been shown in rats challenged with 2,3,7,8-tetrachlorodibenzo-p-dioxin, as demonstrated by the examination of histopathological and ultrastructural images of hippocampus areas characterized by decreased neurodegeneration and smaller inflammatory reactivity [66]. In particular, acetylsalicylic acid was able to decrease the degenerative changes, inflammatory reactivity, as well as the expression of estrogen receptors (atrophy). Older human immunodeficiency virus (HIV)-1 transgenic rats represent a model for HIV-1-associated neurocognitive disorders. In these rats, the chronic low-dose acetylsalicylic acid reduced brain arachidonic acid-metabolite markers of neuroinflammation and oxidative stress (15-epi-lipoxin A4 and 8-isoprostane, PGE2 and leukotriene B4) [67]. In a recent study carried out by Yi et al., the administration of acetylsalicylic acid in combination with statin lowered neurological deterioration as well as platelet aggregation and platelet-leukocyte aggregate numbers in patients after acute ischemic stroke [68]. In a different in vivo study, acetylsalicylic acid (30 mg/kg i.p.) significantly improved learning and memory measured by Morris water maze in ischemic animals (transient middle cerebral artery occlusion (MCAO)) [69]. Moreover, infarction volume and neural changes were significantly counteracted by salicylic acid administration. The normalization of brain function has also been attributed to the treatment with acetylsalicylic acid; this phenolic compound enhanced the inhibition of the allostimulatory capacity of dendritic cells mediated by rapamycin, also reducing the number of mouse bone marrow-derived immature dendritic cells expressing CD40 protein and major histocompatibility complex class II (MHC II) molecules after LPS stimulation [70]. Acetylsalicylic acid has also shown the ability to modulate microglia (BV-2 cells) activity [71]. In these cells, acetylsalicylic acid decreased the expression of transferrin receptor 1 (TfR1), while it up-regulated ferroportin 1 (Fpn1) and ferritin expressions. The authors also demonstrated a higher expression of TfR1 and Fpn1, while ferritin contents, IL-6, TNF-α, and hepcidin mRNA levels were lower in cells treated with acetylsalicylic acid plus LPS compared to cells stimulated with LPS only.

### 4.7. Tannic Acid

Tannic acid is a polyphenol that has shown a highly efficient metal chelating activity. Tannic acid administration to male Wistar-albino rats (50 mg/kg/day for 16 weeks) was able to elevate the concentrations of NR2A and NR2B, two subunits of NMDA receptors, also increasing the activity of antioxidant enzymes such as GPx and decreasing lipid peroxidation measured through MDA at hippocampal level [72]. Tannic acid, administered at the same dosage, also alleviated lead acetate-induced neurochemical perturbations in rat brain [73]. Neuroprotective efficacy of tannic acid on lead acetate-treated rats was exerted through the restoration of the antioxidant status (GSH levels) and antioxidant enzymes’ activity (GST, glutathione reductase (GR), GPx, and SOD), the reduction of oxidative stress (TBARS), and the decrease in neurotoxicity biomarkers enzymes (acetylcholinesterase (AChE), MAO, and Na^+^/K^+^ ATPase). The therapeutic potential of tannic acid has also been validated in an animal model of ischemia/reperfusion injury [74]. Tannic acid significantly reduced oxidative stress, as indicated by the decreased levels of ROS and MDA, whereas increased SOD and nuclear respiratory factor-1 (NRF-1) levels in brain tissues of rats with brain ischemia. The ability of tannic acid to positively modulate behavioral deficits and neurodegeneration has been shown in a rat model of MCAO [75]. MCAO animals pre-treated with tannic acid showed a marked reduction in infarct size, improved neurological function, and suppressed neuronal loss with a down-regulation of GFAP expression. The depletion of the activity of antioxidant enzymes (GPx, GR, GST, glucose-6-phosphate dehydrogenase, SOD, and CAT) and the content of GSH in the MCAO group were protected significantly in the MCAO group pretreated with tannic acid, while the levels of TBARS and pro-inflammatory cytokines were decreased.

### 4.8. Protocatechuic Acid

Protocatechuic acid is a dihydroxybenzoic acid, representing a major metabolite of antioxidant polyphenols found in green tea. Different studies have shown the ability of this phenolic acid to protect PC12 cells against different insults. Protocatechuic acid protected PC12 cells against the toxicity (apoptosis) induced by H_2_O_2_, also increasing GSH levels and CAT activity [76]; in a different study, this phenolic acid suppressed MPP+-induced mitochondrial dysfunction and apoptotic cell death through the counteraction of the loss of mitochondrial membrane potential, formation of ROS, GSH depletion, activation of caspase-3, and down-regulation of Bcl-2 [77]. Protocatechuic acid exerted similar neuroprotective activity in PC12 cells challenged with ROT [78]. As shown by Zhang et al., the neuroprotective activity of protocatechuic acid has also been demonstrated in vivo; in fact, this phenolic acid, in combination with chrysin, prevented neuronal loss in both zebrafish and mice treated by 6-hydroxydopamine (6-OHDA) [79]. In the same study, the combination of protocatechuic acid and chrysin increased cell viability, up-regulated the expression of Nrf2 and of antioxidant enzymes (HO-1, SOD, CAT), decreased oxidative stress (MDA and iNOS), and inflammation (NF-κB) in 6-OHDA-treated PC12 cells. Protocatechuic acid was also able to modulate NF-κB along with MAPK signaling pathways, decreasing pro-inflammatory markers (TNF-α, IL-6, IL-1β, and PGE2), in LPS-treated BV2 microglial cells [80]. The administration (50 and 100 mg/kg) of protocatechuic acid exerted glycemic control, attenuated brain mitochondrial dysfunction, and contributed to the prevention of brain oxidative stress in STZ-induced diabetic rats [81]. Protocatechuic acid protected cerebellar granule neurons from oxidative stress induced by H_2_O_2_ and from nitrosative stress induced by sodium nitroprusside [82]. Neuroprotective effects of protocatechuic acid orally administered (100 mg/kg) in mice treated with sodium arsenate (5 mg/kg) have also been shown [83]. Protocatechuic acid pre-treatment (4 h) before arsenic administration resulted in a decrease in oxidative stress and inflammation with lower levels of lipid peroxidation, iNOS, and NO. The pre-treatment also attenuated arsenic-associated histopathological changes observed at brain level. In a cell-free study, protocatechuic acid was demonstrated to inhibit the aggregation of Aβ and α-Syn, also destabilizing their preformed fibrils [84], then preventing PC12 cell death induced by Aβ and α-Syn.

### 4.9. p-coumaric Acid

Coumaric acid is a molecule part of the polyphenol group widely found in plant-based foods [85]. This phenolic compound has been shown to possess neuroprotective activity both in vitro and in vivo. A study carried out by Guven et al. demonstrated that coumaric acid treatment after ischemia/reperfusion in rat sciatic nerves (SNI) reduced oxidative stress and axonal degeneration [86]. In particular, a significant decrease in MDA paralleled by increased levels of NRF-1 and SOD activity were observed in SNI animals receiving coumaric acid (i.p. single-dose, 100 mg/kg body weight (b.w.)) compared to SNI animals. Additionally, a significantly reduced ischemic fiber degeneration as well as Aβ protein accumulation were observed in rats treated with coumaric acid. Immunohistochemical staining analysis also demonstrated a significant decrease in hypoxia-inducible factor 1-alpha (HIF1α) and NF-κB immunopositive neurons in spinal cord ischemia/reperfusion injury in rats treated with coumaric acid compared with ischemic animals [87]. Neuroprotective effects of the same dosage of p-coumaric acid have also been observed in a rat model of embolic cerebral ischemia [88]. The ability of p-coumaric acid to decrease oxidative damage, focal ischemia, and neurological deficit scores in the brain of rats characterized by cerebral ischemia is attributable to its antioxidant (decreased MDA) and anti-apoptotic (decreased caspase-3) activity. A recent study by Sakamula and Thong-Asa showed that the pre-treatment of animals with p-coumaric acid (100 mg/kg for 2 weeks before inducing the injury) prevented ischemia reperfusion-induced brain oxidative stress (decreased MDA, increased activity of CAT and SOD), infarction size, and hippocampal neuronal death in cerebral ischemia reperfusion injuries [89]. A very recent paper demonstrated that p-coumaric acid mitigates LPS-induced brain damage by decreasing oxidative stress (MDA levels), increasing SOD and GSH levels, and decreasing AChE activity in the brains of mice. This phenolic acid also lowered the production of TNF-α and IL-6 and suppressed neuronal apoptosis by decreasing caspase-3 and c-Jun levels [90]. Neuroprotective effects of p-coumaric acid were also observed in SH-SY5Y cells and primary rat cortical neurons challenged with corticosterone [91]. In these cells, p-coumaric acid effects are attributable to increased SOD and CAT activity as well as to increased CREB phosphorylation mediated by ERK1/2, Akt, and mammalian target of rapamycin (mTOR) pathways.

### 4.10. Sinapic Acid

Sinapic acid, also known as sinapinic acid, is a small naturally occurring hydroxycinnamic acid that can be found in different herbal materials such as berries, oil seeds, and cereals [92]. Sinapic acid has been shown to exert a neuroprotective activity in different animal models of neurodegenerative disorders such as AD and PD. When sinapic acid (10 mg/kg/day, p.o.) was used in a mouse model of AD obtained by bilateral injection of Aβ into the hippocampus, it was able to rescue neuronal cell death at the CA1 region level and attenuate iNOS and nitrotyrosine expression as well as glial cell activation [93]. In the same study, sinapic acid administration, taking place for 1 week beginning immediately after Aβ injection, significantly attenuated memory impairment. A different study was conducted to evaluate whether sinapic acid could exert a beneficial role in an experimental model of early PD represented by unilateral intrastriatal 6-OHDA-lesioned rat [94]. The administration of sinapic acid at the dose of 20 mg/kg b.w. improved turning behavior, counteracted the loss of dopaminergic neurons at substantia nigra pars compacta level, lowered iron reactivity, and attenuated oxidative stress measured as MDA and nitrite levels in midbrain homogenate. Since sinapic acid has shown both a γ-aminobutyric acid type A (GABA(A)) receptor agonistic property and free radical scavenging activity, its potential neuroprotective effects were examined in a global cerebral ischemia animal model (four vessel occlusion model-induced ischemia) [95]. Sinapic acid (10 mg/kg) administered i.p. to rats after induction of ischemia prevented neuronal damage and reduced memory impairment measured by Morris water maze test. The oral administration (10 mg/kg) of this acid was also able to prevent neuronal damage (CA1 and CA3 hippocampal regions) in mice challenged with kainic acid. This neuroprotective activity was accompanied by a reduction of reactive gliosis, iNOS expression, and nitrotyrosine formation in the hippocampus as well as by anticonvulsant and memory-enhancing effects [96].

### 4.11. Ellagic Acid

Ellagic acid is an organic heterotetracyclic phenolic compound obtained from grains and fruits, showing antioxidant activity and known to modulate several intracellular signaling pathways in humans [97]. In a study carried out by Jha et al., ellagic acid showed safety in vitro (SH-SY5Y cells) and neuroprotective activity in vivo (STZ-induced sporadic AD model). Oral administration of ellagic acid at the dose of 50 mg/kg for 30 days significantly reduced the STZ-induced biochemical aberrations, including oxidative stress (decreased MDA and increased GSH and CAT levels), pro-inflammatory markers (GFAP and C-reactive protein (CRP)), and AchE and Aβ plaque levels. Ellagic acid also improved synaptic connectivity, as demonstrated by the elevated level of synaptophysin paralleled by an intact neural architecture. Lastly, the treatment with this phenolic compound normalized the behavioral abnormalities induced by STZ corresponding to irregular spontaneous alternation, reduced locomotor behavior, and memory decline. Beneficial effects of ellagic acid administration have also been observed in the case of animal models of scopolamine- and diazepam-induced cognitive impairments without altering the animals’ locomotion [98]. Two different doses (30 and 100 mg/kg) of ellagic acid were able to significantly revert the amnesia induced by scopolamine in the elevated plus maze and passive avoidance tests in mice, while the same doses antagonized the amnesia diazepam-induced in rats. Moreover, the chronic administration (10 consecutive days) of the lower dosage (30 mg/kg) of this heterotetracyclic phenolic compound ameliorated the memory in rats challenged with diazepam. In a research study carried out by Liu et al., ellagic acid improved endogenous neural stem cells proliferation and neurorestoration through the activation of the Wnt/β-catenin signaling pathway both in vivo and in vitro [99]. Ellagic acid was administered intragastrically in a photothrombosis-induced rat model of brain injury for 7 consecutive days post-venous ischemia, improving nerve-related abilities and decreasing infarct volume and morphological changes at brain level, as well as enhancing the nestin content in the brain’s semidarkness zone. The positive effects coming from the treatment for 2 days with ellagic acid were observed in an oxygen-glucose deprivation and re-perfusion (OGD/R) model of neural stem cells, in which the increased proliferation and expression of both β-catenin and Cyclin D1 at gene level were observed. As showed for sinapic acid, ellagic acid exerted neuroprotection in a PD animal model. In particular, ellagic acid (50 mg/kg b.w./2 mL, by gavages) restored the locomotion and reduced the levels of neuroinflammatory biomarkers (TNF-α and IL-1β) in both the striatum and hippocampus of the rat model of PD obtained by injection of 6-OHDA (right medial forebrain bundle-lesioned rats) [100]. In a more recent publication, it has been shown how the oral administration of ellagic acid exerts neuroprotection against neonatal hypoxic brain injury through the inhibition of inflammatory mediators (e.g., NF-κB p65) and down-regulation of c-Jun N-terminal kinase (JNK)/p38 MAPK activation, also reducing infarct size, weight, and volume of the brain [101].

### 4.12. Salicylic Acid

Salicylic acid is a beta-hydroxy acid derivative of benzoic acid with anti-inflammatory, antibacterial, and neuroprotective activity that occurs as a natural compound in plants. In a methamphetamine (METH)-induced mouse model characterized by dopamine depletion, salicylic acid at both 50 and 100 mg/kg doses has shown the ability to scavenge ROS, revert mitochondrial dysfunction, ameliorate complex-I activity, and decrease neurotoxicity [102]. In this animal model, salicylic acid was also able to block METH-induced behavioral changes related to movement abnormalities. Paclitaxel and cisplatin are two well-known chemotherapeutic drugs often used in combination for the treatment of different solid tumors. Despite that, their use has been related to moderate or, in the worst scenario, severe neurotoxic effects. In primary cortex neurons obtained from Sprague–Dawley rat brains, salicylic acid counteracted the neurotoxicity induced by paclitaxel and cisplatin by exerting a marked antioxidant activity (increased total antioxidant capacity and decreased total oxidant status) [103].

### 4.13. Homovanillic Acid

Homovanillic acid, also known as 3-methoxy-4-hydroxyphenylacetic acid, is a monocarboxylic acid representing a major catecholamine metabolite produced by the action of MAO and COMT as a final product of dopamine metabolism [104]. In a 4-weeks, double-blind, randomized, placebo-controlled study involving a total of 47 subjects (22 men and 25 women), the increase in plasma homovanillic acid (>10%) following cocoa extract (1.4 g/day) consumption along with the improvement of the peripheral dopaminergic activity have been associated with the alleviation of depressive symptoms in overweight or obese adults [105]. In a different human study, the possible associations between changes regarding the levels of plasma homovanillic acid, the severity of psychotic symptoms as well as the improvement in psychotic subjects were investigated [106]. Plasma homovanillic acid levels were measured in patients with psychosis spectrum disorders before and after treatment with first- or second-generation antipsychotics for 6 weeks. Among the 58 subjects that fulfilled all inclusion criteria, 12 had first-episode psychosis (FEP). Data obtained in this study showed that changes in plasma homovanillic acid levels may have a predictive value for responsiveness and possible outcome in individual patients. With respect to FEP patients and patients with (chronic) relapsing psychoses, a significant difference in the decrease in plasma homovanillic acid was observed. This difference correlated with a symptomatic improvement and reaching remission status most pronounced in FEP patients. It was concluded that neuronal plasticity, expressed as changes in plasma homovanillic acid and reflecting the capacity to alter dopamine status, may be crucially involved in the individual responsiveness to antipsychotic treatment, especially in reaching the remission status. In a cohort study carried out by Neider et al., it was investigated the association between the levels of 5-hydroxyindoleacetic acid and homovanillic acid in cerebrospinal fluid (CSF), bullying during childhood, and later suicide in patients suffering from schizophrenia. It was observed that bullying during childhood and a low quotient of homovanillic acid/5-hydroxyindoleacetic acid in CSF are associated with later suicide [107]. With regard to in vitro studies on the neuroprotective activity of homovanillic acid, to the best of our knowledge, no findings are currently available.

### 4.14. Syringic Acid

Syringic acid is a naturally occurring phenolic compound and dimethoxybenzene that can be found in several plants including Ardisia elliptica and Schumannianthus dichotomus [108]. Different preclinical studies have shown the neuroprotective activity of this compound, especially against ischemic conditions. An in vivo study by Güven et al. demonstrated that syringic acid treatment (10 mg/kg b.w., i.p.) in a cerebral ischemia model induced by artery occlusion reduced oxidative stress and neuronal degeneration by increasing the activity of SOD and the levels of NRF-1, while reducing MDA, caspase-3, and caspase-9 levels [109]. The neuroprotective effects of syringic acid were also investigated on spinal cord ischemia injury in rats. Syringic acid pretreatment (10 mg/kg b.w., i.p.) exerted a significant neuroprotective activity characterized by the reduction of the number of apoptotic neurons along with reduced beclin-1 protein levels and caspase-3-immunopositive neurons [110]. Besides these, NRF-1 and SOD activity as well as neurological deficit scores were ameliorated by syringic acid pretreatment. The neuroprotective activity of syringic acid was also demonstrated in an in vitro model of cerebral ischemia established by subjecting hippocampal neurons to OGD/R [111], in which LDH leakage from cells, the expression of both Bax and caspase-3, as well as the intracellular levels of MDA, total ROS, and Ca(2+), were significantly reduced. The above-mentioned changes were paralleled by the increase in JNK phosphorylation, p38 phosphorylated expression, and cell viability, and the rescue of the intracellular SOD, mitochondrial membrane potential, and Bcl-2 expression. Very recently, it was published a study revealing the regulation of mitochondrial biogenesis and energy metabolism by syringic acid, beyond its antioxidant role in the diabetic rats’ brain and spinal tissues [112]. In particular, Rashedinia et al. demonstrated that diabetic (STZ-induced) rats treated with syringic acid administered once per day (100 mg/kg) orally using an intragastric gavage for a period of 6 weeks, exhibited improved learning, memory, and movement deficiency; the administration of this phenolic compound also significantly up-regulated the brain mRNA expression of peroxisome proliferator-activated receptor gamma coactivator 1-alpha (PGC-1α) and NRF-1, both playing a crucial role in the regulation of energy metabolism, oxidative phosphorylation, and mitochondrial biogenesis. Lastly, syringic acid treatment increased the mtDNA/nDNA ratio in the brain as well as in the spinal cord of diabetic rats, also attenuating lipid peroxidation, inflammation, and demyelination in sciatic nerves. During the same year, Ogut et al. evaluated the effect of syringic acid (25 mg/kg/day, oral gavage) on dopamine expression by using behavioral tests related to short-term and recognition memory in Wistar rats [113]. Their results showed that syringic acid was able to increase dopamine along with the percent alternation, time spent in the novel arm, and frequency of novel arm entries of the rats after the Y-maze test. In addition to these effects, syringic acid ameliorated both discrimination index and exploration time in the novel object recognition (NOR) test, and increased the short-term and recognition memory in behavioral tests. A very recent paper showed the neuroprotective effects of syringic acid against AlCl_3_-induced oxidative stress-mediated neuroinflammation in a rat model of AD characterized by reduced memory and learning impairments [114]. The AD rats were supplemented with two doses of syringic acid (25 and 50 mg/kg) for 30 days with an amelioration of the neurobehavioral impairments paralleled by the decreased expression of NF-κB, IL-1β, IL-6, and TNF-α. Syringic acid has also shown the ability to protect retinal ganglion cells against H_2_O_2_-induced apoptosis through the activation of phosphoinositide 3-kinase (PI3K)/Akt signaling pathway [115].

### 4.15. Cinnamic Aldehyde

Cinnamic aldehyde, also referenced as cinnamaldehyde, is a phenylpropanoid organic compound synthesized by the shikimate pathway, occurring naturally as predominantly the *trans* isomer, that gives its distinctive flavor and odor to cinnamon [116]. The neuroprotective effects of cinnamic aldehyde have been reported in different neurodegenerative diseases, including PD. In an in vivo study employing a 1-methyl-4-phenyl-1,2,3,6-tetrahydropyridine (MPTP) mouse model, the selective dopaminergic neuronal death at substantia nigra level was prevented by the administration of this phenylpropanoid compound (10 mg/kg/day, i.p., for 1 week after MPTP injection) [117]. Cinnamic aldehyde protected against MPTP-induced dopaminergic cell death and inhibited the autophagy (down-regulation of p62) stimulated in the substantia nigra of MPTP-treated mice. In the same study the authors, by performing in vitro experiments, were able to show that cinnamic aldehyde recovered MMP+-induced cell death in BE(2)-M17 Cells, also decreasing autophagy, confirming what was observed in vivo. In a different in vivo study, the potential therapeutic effects of cinnamic aldehyde on cerebral ischemia using a mouse model with permanent MCAO were investigated [118]. Mice were treated i.p. with three different doses (25, 50, or 75 mg/kg) of cinnamic aldehyde immediately after cerebral ischaemia, reducing neurological deficit scores, brain edema, and infarct volume; additionally, cinnamic aldehyde was able to inhibit the activation of TLR4, tumor necrosis factor receptor-associated factor 6 (TRAF6), and NF-κB, counteract the increased levels of TNF-α, IL-1β, C-C motif chemokine ligand 2 (CCL2), and endothelial-leukocyte adhesion molecule-1, also reducing the infiltration of leukocytes into the ischaemic brain. Gürer et al. have recently demonstrated that this aldehyde can provide neuroprotection as well as attenuation of cerebral vasospasm after subarachnoid hemorrhage in rabbits [119]. The i.p. administration of cinnamic aldehyde (5 min; 50 mg/kg/day), for a total of 3 days following an intracisternal blood injection, significantly increased the cross-sectional areas of the basilar artery, also decreasing both arterial wall thickness and hippocampal degeneration scores. In an in vitro study carried out by Emamghoreishi and collaborators the neuroprotective effects of cinnamic aldehyde against Aβ inSH-SY5Y cells and the contribution of NMDA, ryanodine, adenosine receptors, and GSK-3β were investigated [120]. The results showed that the treatment with cinnamic aldehyde significantly reverted Aβ-induced toxicity, while adenosine, NMDA, and dantrolene (a ryanodine receptor antagonist) inhibited the neuroprotection exerted by this natural compound. Some of the concentrations of cinnamic aldehyde used were also able to suppress the Aβ-induced activation of GSK-3β. METH represents an illegal drug able to markedly stimulate the central nervous system and induce degeneration of dopaminergic and serotonergic axons. The challenge of PC-12 cells with METH (2.5 mM) decreased the cell viability and GSH levels, increased ROS, and induced apoptosis [121]. Notably, the pre-treatment with *trans*-cinnamic aldehyde significantly attenuated all the alterations METH-induced. The combination of the *trans* form of this aldehyde with ellagic acid has also shown the potential to alleviate aging-induced cognitive impairment through the modulation of mitochondrial function (ROS, mitochondrial membrane potential, and ATP level) and inflammatory (TNF-α and IL-1β) and pro-apoptotic (Bax and caspase-3) mediators in the prefrontal cortex of aged rats [122]. The therapeutic and neuroprotective potential of cinnamic aldehyde (100 mg/kg) has shown to decrease neutrophil recruitment, suppress ROS, reduce histologic damage and acute hippocampal dysfunction (Y-maze test) in a TBI rat model [123]. A study performed by Etaee et al. investigated the possible anxiolytic effects of the administration of cinnamic aldehyde (20 mg/kg/day for 7 days) on male Swiss mice subjected to acute or chronic stress [124]. By using elevated plus maze and open field behavioral tests, the authors were able to demonstrate how cinnamic aldehyde could decrease anxiety-related behavior in mice.

Table 1 reports the names of each phenolic acid considered, their basic description, the effects on different disease models, and the possible mechanism of action as well as the chemical structure.

Table 1 does not include the findings related to homovanillic acid, a methoxyphenol for which only human studies not describing the mode of action related to neuroprotection were reported.

### 4.16. Phenolic Acids Potential in Drug Discovery

Natural products, including phenolic acids, represent an important and wide source of new lead compounds in drug discovery and development. Unfortunately, the discovery of potential “hits” (lead molecules) is a complex and very challenging process, requiring the evaluation of numerous parameters during drug candidate selection (e.g., safety, pharmacokinetics, and efficacy) along with a considerable economic effort and a large workforce [125]. Among compounds of natural origin, phenolic acids, due to their phenol moiety coupled to the resonance-stabilized structure, possess a strong antioxidant activity often accomplished via a radical scavenging mechanism. The antioxidant activity together with those previously mentioned such as anti-inflammatory and anti-aggregant activities (see Table 1), make phenolic acids an excellent starting point for the synthesis of new drugs characterized by a multimodal mechanism of action. Different research groups are currently employing conventional and innovative drug discovery strategies such as microfluidics [126] and computer-aided drug design [125] to isolate and elucidate the structure of natural phenolic acids with prominent biological activity, to study the structure-activity-relationship (SAR) of novel phenolic acids characterized by a high pharmacological potential [127], and to synthesize new bioactive molecules based on naturally occurring phenolic acids and/or their derivatives [128].

## 5. Conclusions

In conclusion, recent evidence suggests that phenolic acids may exert neuroprotective effects targeting multiple cellular pathways involved in the pathophysiology of cognitive disorders. Since the results from in vivo reports are an indicator of potential mechanisms, it is important that future studies would be able to replicate the findings at the clinical level using food sources of phenolic acids or nutraceutics with clinically relevant doses. Moreover, future studies should also take into account that phenolic acids undergo metabolism and transformation by the gut microbiota into bioavailable molecules, whose effects and effectiveness may differ. Although the pharmacokinetic properties of phenolic acids are considerable, and they can easily reach the brain at therapeutically-relevant levels, future translational studies should also investigate which among the different metabolites generated from phenolic acid consumption may be responsible for the potential neuroprotective effects of this group of polyphenols in the treatment of cognitive disorders.

## Figures and Tables

**Table 1:** Phenolic acids and related characteristics.

Phenolic Acids	Basic Description	Disease Models	Mode of Action	Ref.	Chemical Structure
*Caffeic acid*	Organic compound classified as a hydroxycinnamic acid. It consists of both phenolic and acrylic functional groups. Since it represents an intermediate in the biosynthesis of lignin (one of the principal components of woody plant biomass and its residues), caffeic acid can be found in all plants.	Mice treated with LPS	▪ Attenuated sickness behavior ▪ Decreased oxidative stress ▪ Decreased inflammation	[18]	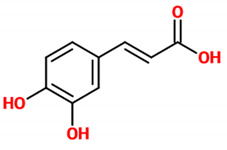
		Human α-syn aggregation	▪ Counteracted aggregation	[21]	
		Mouse model of epilepsy	▪ Decreased oxidative stress and DNA damage	[22]	
		Mouse model of acute seizure (diazepam and aspilocarpine-induced)	▪ Reduced the latency to sleep ▪ Reduced genotoxic damage	[23]	
		Rat model of hyperinsulinemia	▪ Modulated cerebral insulin signaling, Aβ accumulation, and synaptic plasticity ▪ Ameliorated memory and learning impairments ▪ Enhanced the antioxidant defense ▪ Decreased the expression of p-tau in the hippocampus ▪ Attenuated the expression of APP and β-site APP cleaving enzyme ▪ Increased the expression of synaptic proteins	[24]	
*Caffeic acid phenethyl ester*	Ester of caffeic acid and phenethyl alcohol.	Rats exposed to ionizing radiation	▪ Reduced oxidative stress ▪ Ameliorated the antioxidant defense	[25]	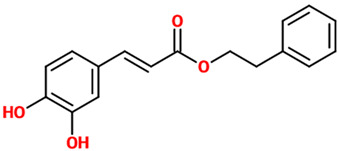
		Rats treated with IFOS	▪ Decreased oxidative status and protein carbonyl levels	[28,29]	
		Mouse model of HD (3-nitropropionic acid-induced)	▪ Reduced striatal damage and the behavioral deficits ▪ Reduced the activation of astrocyte and microglia	[31]	
		BV-2 cells treated with LPS	▪ Reduced oxidative stress ▪ Attenuated LPS-dependent MAPK and Akt signaling pathways ▪ Attenuated induction of HO-1 and EPO	[27]	
		PC12 cells treated with (MPP+)	▪ Increased the network of neuritis ▪ Increased the expression of proteins responsible for axonal growth and synaptogenesis	[30]	
		Rat cerebellar granule neurons treated with SNP or glutamate/glycine or H_2_O_2_	▪ Decreased nitrosative stress, excitotoxicity, and oxidative stress	[38]	
*Chlorogenic acid*	Ester of caffeic acid and (−)-quinic acid. It belongs to the polyphenol family of esters, including hydroxycinnamic acids (caffeic acid, ferulic acid and p-coumaric acid) with quinic acid.	Cortical mouse neurons treated with L-glutamic acid	▪ Regulated the intracellular concentrations of Ca(2+)	[32]	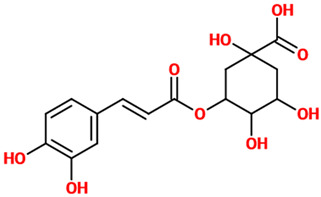
		Microglia infected with herpes simplex virus	▪ Decreased inflammation ▪ Increased the survival rate ▪ Prevented the increase in TLR2, TLR9, and Myd88	[33]	
		Rats treated with H_2_O_2_	▪ Reduced oxidative stress	[35]	
		Mouse model of epilepsy (pilocarpine-induced)	▪ Decreased lipid peroxidation ▪ Decreased nitrosative stress ▪ Reduced mRNA expression levels of NMDA receptors and mGluR1/mGluR5	[36]	
		PC12 cells treated with ethanol	▪ Increased the cell viability and promoted the proliferation of damaged cells ▪ Increased the distribution ratio of the cells at the G2/M and S phases ▪ Enhanced mitochondrial transmembrane potential ▪ Modulated apoptosis mediators	[37]	
		Rat cerebellar granule neurons treated with SNP	▪ Protected against NO effects	[38]	
*Ferulic acid*	Natural phenylpropanoid found in Euphorbia hylonoma herbs. It is a substituted derivative of *trans*-cinnamic acid.	N/A (untreated mice)	▪ Decreased inflammation ▪ Decreased oxidative stress ▪ Increased serotonin and norepinephrine levels ▪ Reduced depressive-like behavior ▪ Inhibited the activity of MAO-A	[39]	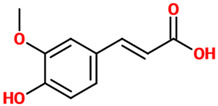
		N/A (untreated mice)	▪ Improved antidepressant-like effect ▪ Increased antioxidant machinery in the cerebral cortex of mice	[40]	
		Rat model of PD (ROT-induced)	▪ Improved antidepressant-like effect ▪ Increased antioxidant machinery in the cerebral cortex of mice	[41]	
		Microglial cells treated with LPS	▪ Decreased inflammation ▪ Decreased oxidative stress	[42]	
		Neuro-2a cells treated with H_2_O_2_	▪ Antioxidant and anti-inflammatory activity ▪ Up-regulated BDNF ▪ Modulated apoptosis mediators	[43]	
		Mouse model of chronic unpredictable mild stress	▪ Up-regulated BDNF, PSD95, and synapsin I levels	[44]	
		PC12 cells treated with LPS	▪ Decreased inflammation ▪ Attenuated the up-regulation of phosphodiesterase 4 activity ▪ Decreased the up-regulation of the PDE4B mRNA ▪ Reverted the down-regulation of CREB and pCREB	[45]	
		Rat model of focal cerebral ischemic injury	▪ Down-regulated MEK/ERK/p90RSK signaling pathway	[46]	
		Hypoxia-stressed PC12 cells	▪ Increased cell viability ▪ Prevented membrane damage ▪ Decreased oxidative stress ▪ Decreased intracellular free Ca(2+) levels, lipid peroxidation, and PGE2 production ▪ Reduced p-p38 MAPK ▪ Modulated apoptosis mediators	[47]	
*Gallic acid*	Phenolic acid classified as trihydroxybenzoic acid. It is found in gallnuts, sumac, witch hazel, tea leaves, oak bark, and in several other plants.	Mice, Neuro-2A, and primary microglial cells treated with Aβ	▪ Counteracted cognitive dysfunction and down-regulated the levels of NF-κB acetylation in mice ▪ Increased Neuro-2A cells viability ▪ Decreased inflammation in microglia	[48]	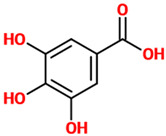
		Rat model of TBI	▪ Prevented memory deficits and synaptic impairment ▪ Decreased inflammation	[49]	
		Transgenic mice model of AD (APP/PS1)	▪ Rescued learning and memory deficits	[50]	
		Transgenic mice model of AD (APP/PS1)	▪ Improved cognition ▪ Counteracted synaptic impairment ▪ Reduced Aβ aggregation	[53]	
		Rat model of TBI	▪ Improved memory and LTP impairment ▪ Decreased lipid peroxidation ▪ Decreased inflammation	[54]	
		Mouse model of diabetes (STZ-induced)	▪ Improved oxidative status	[55]	
		Rats treated with cyclophosphamide	▪ Decreased oxidative stress ▪ Enhanced the antioxidant defense system	[56]	
		Rats treated with sodium arsenite	▪ Improved cognition ▪ Decreased oxidative stress ▪ Enhanced the antioxidant defense system	[57]	
		Rat model of AD (AlCl_3_-induced)	▪ Ameliorated hippocampal neurodegeneration and cognitive impairment ▪ Decreased oxidative stress ▪ Rescued the antioxidant defense system ▪ Restored neurotransmitter levels	[58]	
*Rosmarinic acid*	A polyphenol constituent of many culinary herbs such as rosemary, mint, and basil. From the chemical point of view, it represents a caffeic acid ester, with tyrosine giving another phenolic ring via dihydroxyphenyl-lactic acid.	Kindling mouse model (PTZ-induced)	▪ Reduced the levels of free radicals and DNA damage ▪ Increased the latency ▪ Decreased the percentage of seizure incidents	[22]	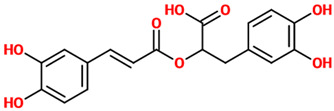
		Kindling mouse model (PTZ and pilocarpine-induced)	▪ Improved the latency to first seizures ▪ Reduced the latency to sleep in the diazepam-induced sleeping time test ▪ Decreased pilocarpine-induced genotoxic damage	[23]	
		Rats exposed to noise	▪ Attenuated hearing loss and hair cell damage ▪ Reduced oxidative stress and lipid peroxidation ▪ Enhanced the antioxidant machinery	[59]	
		C6 glial cells treated with H_2_O_2_	▪ Reduced oxidative stress and lipid peroxidation ▪ Increased cell viability	[60,61]	
		Rat model of SCI	▪ Enhanced the antioxidant status ▪ Decreased oxidative stress ▪ Decreased inflammation	[62]	
		Mouse model of a chronic restraint stress	▪ Decreased p-tau and insoluble p-tau formation ▪ Reverted the abnormal changes of chaperones and Pin1	[63]	
		Rat organotypic hippocampal slice cultures treated with scopolamine	▪ Enhanced LTP ▪ Enhanced BDNF and GluR-2 protein expression ▪ Enhanced cell survival rate	[64]	
		Rat model of neuropathic pain	▪ Decreased inflammation	[65]	
*Acetylsalicylic acid*	A weakly acidic substance widely used as a medication to reduce pain, fever, as well as inflammatory processes. Chemically, it represents an acetyl derivative of salicylic acid.	Rats treated with tetrachlorodibenzo-p-dioxin	▪ Decreased inflammation	[66]	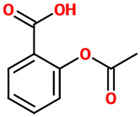
		HIV-1 transgenic rat	▪ Decreased inflammation ▪ Decreased oxidative stress	[67]	
		Rat model of ischemia	▪ Improved learning and memory ▪ Counteracted infarction volume and neural changes	[69]	
		Mouse bone marrow-derived immature dendritic cells treated with LPS	▪ Reduced the number of mouse bone marrow-derived immature dendritic cells expressing CD40 protein and MHCII	[70]	
		BV-2 cells treated with LPS	▪ Enhanced the expression of TfR1 and Fpn1 ▪ Decreased the levels of ferritin contents ▪ Decreased inflammation	[71]	
*Tannic acid*	A specific form of tannins, a class of astringent, polyphenolic biomolecules, characterized by a very efficient metal chelating activity.	N/A (untreated rats)	▪ Elevated the concentrations of NMDA receptors ▪ Enhanced the antioxidant machinery ▪ Decreased lipid peroxidation	[72]	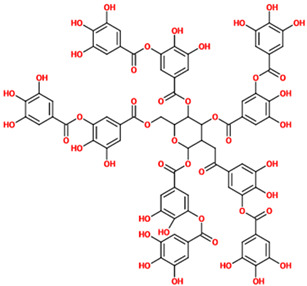
		Rats treated with lead acetate	▪ Decreased the neurochemical perturbations ▪ Decreased oxidative damage ▪ Restored antioxidant status	[73]	
		Rat model of ischemia/reperfusion injury	▪ Decreased lipid peroxidation ▪ Enhanced the antioxidant machinery	[74]	
		Rat model of MCAO	▪ Counteracted behavioral deficits and improved neurological function ▪ Decreased neurodegeneration ▪ Reduced infarct size	[75]	
*Protocatechuic acid*	A dihydroxybenzoic acid representing a major metabolite of antioxidant polyphenols found in green tea. It also possesses anti-inflammatory properties.	PC12 cells treated with H_2_O_2_	▪ Modulated apoptotic mediators ▪ Enhanced the antioxidant defense	[76]	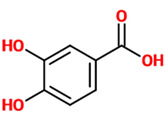
		PC12 cells treated with MPP+	▪ Suppressed mitochondrial dysfunction and apoptotic cell death ▪ Decreased oxidative stress ▪ Enhanced the antioxidant defense	[77]	
		PC12 cells treated ROT	▪ Ameliorated mitochondrial dysfunction ▪ Suppressed apoptotic cell death	[78]	
		Zebrafish, mice, and PC12 treated with 6-OHDA	▪ Protocatechuic acid in combination with chrysin ▪ Prevented neuronal loss in both zebrafish and mice ▪ Increased cell viability, decreased oxidative stress, enhanced the antioxidant machinery, and decreased inflammation in PC12 cells	[79]	
		BV2 cells treated with LPS	▪ Modulated NF-κB and MAPKs signaling pathways ▪ Decreased inflammation	[80]	
		Rat model of diabetes (STZ-induced)	▪ Exerted glycemic control ▪ Attenuated brain mitochondrial dysfunction ▪ Decreased oxidative stress	[81]	
		Cerebellar granule neurons treated with H_2_O_2_ and BV2 cells treated with LPS	▪ Decreased nitrosative stress and neurodegeneration in cerebellar granule neurons ▪ Decreased inflammation in BV-2 cells	[82]	
		Mice treated with sodium arsenate	▪ Decreased lipid peroxidation and oxidative stress ▪ Decreased inflammation ▪ Attenuated histopathological changes	[83]	
		PC12 cells treated with Aβ and α-Syn	▪ Prevented cell death ▪ Inhibited the aggregation of Aβ and α-Syn	[84]	
*p-coumaric acid*	A hydroxycinnamic acid representing the hydroxy derivative of cinnamic acid. Among the three isomers of coumaric acid (o-, m-, and p-coumaric acid), p-coumaric acid represents the most abundant isomer that can found in nature.	Rat model of SNI	▪ Reduced oxidative stress and axonal degeneration ▪ Enhanced the antioxidant defense	[86]	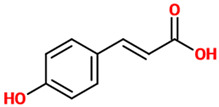
		Rat model of SCI	▪ Reduced ischemic fiber degeneration ▪ Reduced Aβ protein accumulation ▪ Decreased neuroinflammation	[87]	
		Rat model of embolic cerebral ischemia	▪ Modulated apoptotic mediators ▪ Decreased lipid peroxidation	[88]	
		Rat model of ischemia/reperfusion injury	▪ Decreased lipid peroxidation ▪ Enhanced the antioxidant defense ▪ Decreased infarction size and hippocampal neuronal death	[89]	
		Mice treated with LPS	▪ Modulated apoptotic mediators ▪ Decreased lipid peroxidation	[90]	
		SH-SY5Y cells and primary rat cortical neurons treated with corticosterone	▪ Enhanced the antioxidant defense ▪ Increased CREB phosphorylation mediated by ERK1/2, Akt, and mTOR pathways	[91]	
*Sinapic acid*	A small naturally occurring hydroxycinnamic acid belonging to the phenylpropanoid family. Due to its well-known ability to absorb laser radiation and donate protons to the analyte of interest, it is frequently used as a matrix in MALDI mass spectrometry experiments.	Mouse model of AD (Aβ-induced)	▪ Rescued neuronal cell death at CA1 region level ▪ Attenuated oxidative and nitrosative stress ▪ Attenuated memory impairment ▪ Attenuated glial cell activation	[93]	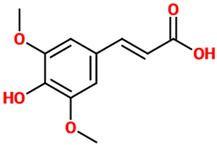
		Rat model of early PD (6-OHDA-induced)	▪ Improved turning behavior ▪ Counteracted the loss of dopaminergic neurons at substantia nigra pars compacta level ▪ Lowered iron reactivity ▪ Attenuated oxidative stress	[94]	
		Rat model of global cerebral ischemia	▪ Prevented neuronal damage ▪ Reduced memory impairment	[95]	
		Mice treated with kainic acid	▪ Prevented neuronal damage ▪ Reduced reactive gliosis ▪ Reduced oxidative and nitrosative stress ▪ Enhanced memory impairments	[96]	
*Ellagic acid*	An organic heterotetracyclic compound found in different fruits and vegetables. From the chemical point of view, it represents the dilactone of hexahydroxydiphenic acid.	Rat model of sporadic AD (STZ-induced)	▪ Reduced oxidative stress ▪ Reduced inflammation ▪ Reduced AchE and Aβ plaque levels ▪ Improved synaptic connectivity ▪ Normalized sporadic AD-associated abnormal behavioral representations	[97]	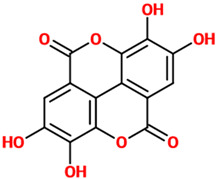
		Rat models of scopolamine- and diazepam-induced cognitive impairments	▪ Prevented scopolamine- and diazepam-induced cognitive impairments	[98]	
		Rat model of nerve injury (photothrombosis-induced) and OGD/R model in neural stem cells	▪ Improved the rats’ nerve-related abilities, remedied infarct volumes and morphological changes in the brain, and enhanced the content of nestin protein in the brain semidarkness zone ▪ Improved cell proliferation and neurorestoration through the activation of the Wnt/β-catenin signaling pathway	[99]	
		Rat model of PD (6-OHDA-induced)	▪ Restored the locomotion ▪ Decreased inflammation in striatum and hippocampus	[100]	
		Rat model of neonatal hypoxic-ischemic brain injury	▪ Reduced infarct size, volume and tissue loss ▪ Decreased neurodegeneration and inflammation ▪ Down-regulated MAPK proteins ▪ Modulated apoptotic mediators	[101]	
*Salicylic acid*	A plant hormone representing a precursor to and a metabolite of acetylsalicylic acid (commonly known as aspirin).	Mice treated with METH	▪ Decreased oxidative stress ▪ Reverted mitochondrial dysfunction and ameliorated complex-I activity ▪ Decreased neurotoxicity ▪ Blocked behavioral changes related to movement abnormalities	[102]	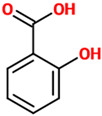
		Primary cortex neurons treated with paclitaxel and cisplatin	▪ Decreased cell death ▪ Increased total antioxidant capacity ▪ Decreased oxidative stress	[103]	
*Syringic acid*	A dimethoxybenzene that is 3,5-dimethyl ether derivative of gallic acid having a role as a plant metabolite. It can be found in several plants such as Ardisia elliptica.	Rat model of brain ischemia injury	▪ Decreased neuronal degeneration ▪ Reduced oxidative stress ▪ Increased total antioxidant capacity ▪ Modulated apoptotic mediators	[109]	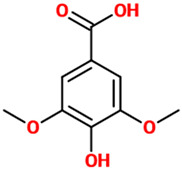
		Rat model of SCI	▪ Decreased neuronal degeneration ▪ Modulated apoptotic mediators ▪ Ameliorated neurological deficit	[110]	
		Hippocampal neurons subjected to OGD/R	▪ Modulated apoptotic mediators ▪ Decreased oxidative stress ▪ Decreased intracellular levels Ca(2+) ▪ Increased the levels of JNK phosphorylation, p38 phosphorylated expression ▪ Increased cell viability ▪ Rescued mitochondrial membrane potential and antioxidant defense	[111]	
		Rat model of diabetes (STZ-induced)	▪ Up-regulated the key regulators of energy metabolism, oxidative phosphorylation, and mitochondrial biogenesis ▪ Attenuated lipid peroxidation ▪ Reduced inflammation and demyelination in sciatic nerves ▪ Improved learning, memory, and movement deficiency	[112]	
		Rats treated with deltamethrin	▪ Increased the dopamine levels ▪ Ameliorated behavioral tests related to short-term and recognition memory	[113]	
		Rat model of AD (AlCl_3_-induced)	▪ Decreased oxidative stress ▪ Decrease inflammation ▪ Ameliorated neurobehavioral impairments	[114]	
		Retinal ganglion cells treated with H_2_O_2_	▪ Inhibited cell injury ▪ Decreased oxidative stress and lipid peroxidation ▪ Modulated apoptotic mediators ▪ Activated PI3K/Akt signaling pathway	[115]	
*Cinnamic aldehyde*	A phenylpropanoid synthesized by the shikimate pathway giving to cinnamon its characteristic flavor and odor. It can be found in the bark of cinnamon trees as well as other species of the genus Cinnamomum.	Mouse model of PD (MPTP-induced) and BE(2)-M17 cells treated with MPTP	▪ Inhibited autophagy and prevented the selective dopaminergic neuronal death in the substantia nigra ▪ Decreased autophagy and recovered MPP+-induced cell death in BE(2)-M17 cells	[117]	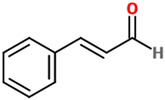
		Mouse model of permanent cerebral ischemia	▪ Reduced neurological deficit scores, brain edema, and infarct volume ▪ Decreased inflammation ▪ Reduced the infiltration of leukocytes into the ischaemic brain	[118]	
		Rabbit model of early brain injury (subarachnoid hemorrhage-induced)	▪ Attenuated cerebral vasospasm ▪ Increased the cross-sectional areas of the basilar artery and reduced the arterial wall thickness ▪ Lowered hippocampal degeneration scores	[119]	
		SH-SY5Y cells treated with Aβ	▪ Reverted cell toxicity ▪ Suppressed the activation of GSK-3β	[120]	
		PC-12 cells treated with METH	▪ Attenuated cell viability loss ▪ Decreased oxidative stress ▪ Restored the antioxidant defense ▪ Modulated apoptotic mediators	[121]	
		Aged rats treated with METH	▪ Attenuated aging-induced memory impairment ▪ Decreased oxidative stress ▪ Decreased inflammation ▪ Modulated apoptotic mediators	[122]	
		Rat model TBI	▪ Decreased neutrophil recruitment ▪ Decreased oxidative stress ▪ Reduced histologic damage and acute hippocampal dysfunction	[123]	
		Mice subjected to acute or chronic stress	▪ Decreased anxiety-related behavior in mice	[124]	

## Data Availability

Not applicable.
